# Protected Health Information on Social Networking Sites: Ethical and Legal Considerations

**DOI:** 10.2196/jmir.1590

**Published:** 2011-01-19

**Authors:** Lindsay A Thompson, Erik Black, W Patrick Duff, Nicole Paradise Black, Heidi Saliba, Kara Dawson

**Affiliations:** ^3^University of FloridaDepartment of MedicineGainesville, FLUnited States; ^2^University of FloridaCollege of EducationGainesville, FLUnited States; ^1^University of FloridaDepartment of PediatricsGainesville, FLUnited States

**Keywords:** Protected health information, medical missions, Internet

## Abstract

**Background:**

Social networking site use is increasingly common among emerging medical professionals, with medical schools even reporting disciplinary student expulsion. Medical professionals who use social networking sites have unique responsibilities since their postings could violate patient privacy. However, it is unknown whether students and residents portray protected health information and under what circumstances or contexts.

**Objective:**

The objective of our study was to document and describe online portrayals of potential patient privacy violations in the Facebook profiles of medical students and residents.

**Methods:**

A multidisciplinary team performed two cross-sectional analyses at the University of Florida in 2007 and 2009 of all medical students and residents to see who had Facebook profiles. For each identified profile, we manually scanned the entire profile for any textual or photographic representations of protected health information, such as portrayals of people, names, dates, or descriptions of procedures.

**Results:**

Almost half of all eligible students and residents had Facebook profiles (49.8%, or n=1023 out of 2053). There were 12 instances of potential patient violations, in which students and residents posted photographs of care they provided to individuals. No resident or student posted any identifiable patient information or likeness in text form. Each instance occurred in developing countries on apparent medical mission trips. These portrayals increased over time (1 in the 2007 cohort; 11 in 2009; *P* = .03). Medical students were more likely to have these potential violations on their profiles than residents (11 vs 1, *P* = .04), and there was no difference by gender. Photographs included trainees interacting with identifiable patients, all children, or performing medical examinations or procedures such as vaccinations of children.

**Conclusions:**

While students and residents in this study are posting photographs that are potentially violations of patient privacy, they only seem to make this lapse in the setting of medical mission trips. Trainees need to learn to equate standards of patient privacy in all medical contexts using both legal and ethical arguments to maintain the highest professional principles. We propose three practical guidelines. First, there should be a legal resource for physicians traveling on medical mission trips such as an online list of local laws, or a telephone legal contact. Second, institutions that organize medical mission trips should plan an ethics seminar prior the departure on any trip since the legal and ethical implications may not be intuitive. Finally, at minimum, traveling physicians should apply the strictest legal precedent to any situation.

## Introduction

Online social networking applications (eg, Facebook, Flickr, Twitter, and YouTube) have become the fastest-growing mechanism to exchange personal and professional information. With 85%-95% of students on college campuses using these communication mediums, and all age groups, even senior citizens, rapidly adopting their use [1,2], online social networking applications have emerged as a significant means of interaction for sharing everything from casual greetings to displaying wedding photographs and lobbying for humanitarian fundraising.

Medical professionals who use social networking sites have unique responsibilities, since their postings could portray themselves in unprofessional ways [3] or, most important, potentially violate patient privacy [3,4]. Publicized breaches of privacy might stem from careless oversights to malicious, illegal, and blatantly unprofessional behaviors. Most worrisome would be those that involve medical students and residents, since their unprofessional behaviors are known to be linked to lifelong licensure problems with state medical boards [5]. Poignantly, Chretien et al recently demonstrated that a significant number of academic medical institutions have experienced incidents of unprofessional student online postings in which some were severe enough to end in student dismissal, although the reasons for these dismissals were not disclosed [6]. Broadly stated, breaches of patient confidentiality involve the identification or potential identification of a patient in any way. Many laws, including the Health Information Portability and Accountability Act (1996, HIPAA), are in place to defend this principle [7,8]. In this context, this study aimed to document whether medical trainees ever share or discuss their patient interactions in their online profiles.

## Methods

**Figure 1:**
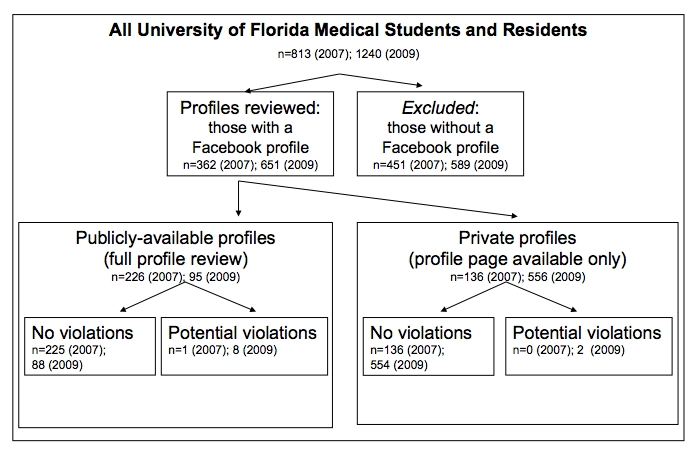
Enrollment of medical students and residents’ Facebook profiles

The University of Florida’s Institutional Review Board approved as exempt a multidisciplinary team to perform two cross-sectional analyses of Facebook profiles of medical students and residents (2007, 2009). For the purposes of this study, we considered eligible all medical students (n = 501 in 2007, 528 in 2009) enrolled at the University of Florida, Gainesville, and the associated medical residents with available full names (n = 312 in 2007, 712 in 2009) employed by the Shands Hospital. Descriptive findings from each cohort have been published elsewhere [3,9]. In brief, Facebook proceedings allow any registered user of Facebook access to every Facebook profile according to each individual owner’s chosen preferences for privacy. To be a Facebook user, an applicant only needs to supply an email address and choose a password. Once a user, one can scan Facebook profiles anonymously, without revealing to the profile owners that their site has been viewed. Drawing on these parameters, the first profile search associated with this study was done from June 7 to June 11, 2007, where three researchers used personally created Facebook accounts to manually search for the study subjects’ online profiles using a university-generated list of names of students and residents. The second cohort was searched from September 2 to October 7, 2009, where only one study author (EB) used a personal account to manually search for the study subjects’ online profiles using the 2009 university lists. Given that the study began in 2007, it did not use any face-recognition software, since it was not available at the time, and our study protocol did not include searching “friends’ sites” for the study subjects. We likewise could not discern how often a profiler used Facebook, nor could we tell the duration that a profiler had the account. We could not discern the frequency with which a subject accessed Facebook. This study was part of a larger study on trends of social networking site use among medical students and residents [3,9]. We first determined whether each student or resident had a Facebook account and whether that account was “private” or “public,” a designation that each user can activate to limit some, or all, of a site’s content. Sites were deemed private if the following message appeared on the site of interest: “_____ only shares certain information with everyone. If you know ____, add him/her as a friend on Facebook.” Three study authors (EB, LT, KD) compared their individual analyses of the content. We found a high degree of interrater reliability using intraclass correlation (type 1, *df* = 6) = 0.9, for the public profiles of medical students and residents in characterizing material with complete unanimity for the comparisons of potential patient privacy violations [9]. We searched a total of 1023 profiles (n = 372 profiles in 2007 and 651 in 2009; see Figure 1 for flowchart of subjects and profiles reviewed).

For the purposes of this study, we reviewed sites for possible privacy violations to explicitly examine how students are using sites according to legal and ethical professional norms. Once a profile related to a study subject was found, any potential violation within a site was counted as one, even if a profile had multiple representations. For private sites, where optional Facebook privacy settings can limit non-“friends” from viewing part or most of the site, study authors reviewed only the profile photograph(s) and available content on their front page, where Facebook users can choose to list information such as name, address, and favorite hobbies. For publicly available profiles (n = 233, 62.6% in 2007; n = 95, 14.6% in 2009), we manually scanned all information, including all scrolled wall posts in text form and extensive albums for photographs, for patient information, such as names, dates, and procedures, photographs of patients or procedures, or any mention of patients. We also recorded available demographic information of the subjects (gender, year in training, relative age of subject). At the end of the study, in September 2010, we reviewed the sites that had potential privacy violations; all sites were now private and could not be reviewed. We performed our analyses using SPSS PASW Statistics, version 17 (Chicago, IL), and we accepted a level of significance of *P* < .05 using a Student *t* test for comparison [10].

## Results

**Figure 2:**
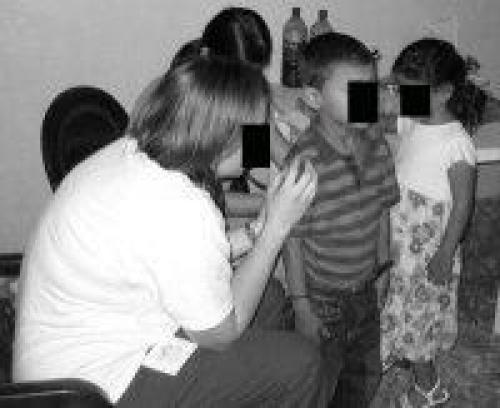
Example of a potential violation of patient privacy

**Figure 3:**
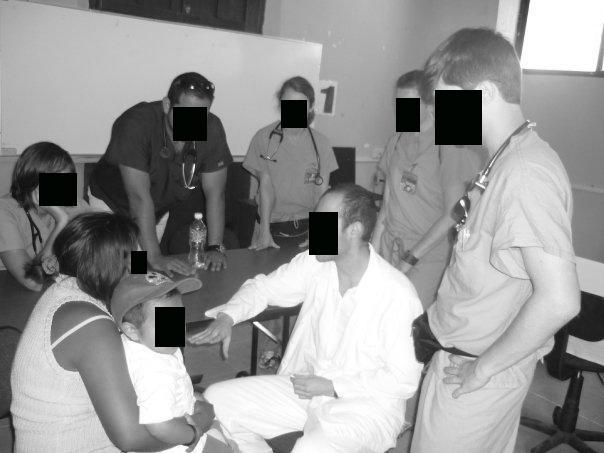
Example of a potential violation of patient privacy

**Figure 4:**
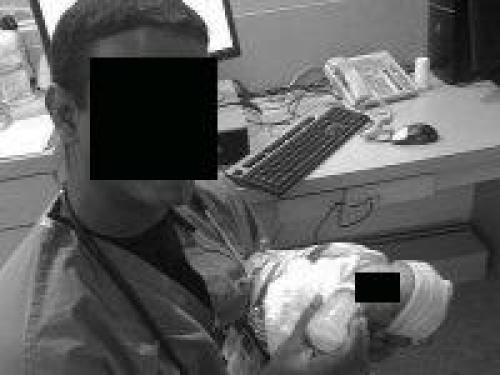
Example of a potential violation of patient privacy

**Figure 5:**
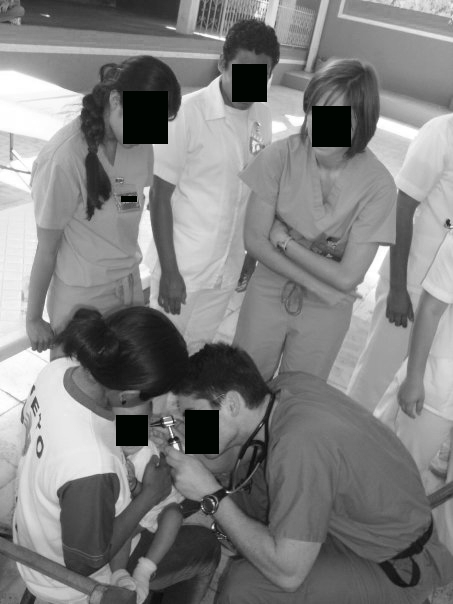
Example of a potential violation of patient privacy

A significant proportion (49.8%) of medical students and residents had profiles (n = 1023 out of 2053 eligible students and residents). Students and residents increased their use of Facebook, with 44.5% using Facebook in 2007 (n = 362 of 813), compared to 52.5% in 2009 (n = 651 of 1240, *P* < .0001). By 2009, a majority (85.4%) of profiles were made private by their owners compared to 37.6% in 2007 (*P* < .001). However, we found significant and increasing evidence of potential privacy violations (n = 12; 1 of 372 in 2007, 10 of 651 in 2009; *P* = .03). Medical students were more likely than residents to have these violations (10 students, 1 resident; *P* = .04). In each instance, all of which were photographic patient information, the profile owners illustrated themselves providing health care to individuals (see deidentified examples, Figures 2-5; authors added the face blockouts). We did not find any textual evidence of patient information or likeness that could potentially violate patient privacy.

In each of these groups of photographs, the profile owner was apparently on a medical mission trip, performing health care in another county. These photographs were placed in photo albums that the profile owner explicitly labeled (eg, “mission trip” or “Dominican Republic”), giving the viewer a context for understanding where they are from. Among “private” profiles (n = 701), two displayed themselves on their profile picture with identifiable patients, which is the information first available on any profile when a user peruses Facebook profiles. For those with publicly available Facebook profiles (n = 328 total, 233 in 2007; 95 in 2009), 10 additional sites had potential privacy violations within their profile’s photo albums. Photographs included trainees interacting with identifiable patients or performing medical examinations or procedures such as vaccinations. Of note, in each photograph, the recipient of the care was a child. 

## Discussion

This study reveals that students and residents place protected health information on their publicly available social networking sites. This exposes significant concerns with the ethical and legal aspects of patient portrayals, a problem well debated with cyberspace issues [11,12], but one that has been magnified by the recent phenomenon of online social networking [13]. As an unanticipated outcome, these violations seem only to be in the context of medical mission trips. Medical missions, defined as a “group of people traveling from a developed country to a developing country for a short period of time” [14] with the purpose of providing needed health care, are viewed as highly professional, benevolent acts [15]. Nonetheless, posting photographs or information from such events challenges US and international laws of patient privacy, regardless of whether content is posted to a publicly available or private profile. Imagery of humanitarian trips is common, even supported in medical settings [15]; perhaps the reason why this online imagery is not only common but increasing. It is likely, given the increasing frequency of these portrayals, that medical students and residents believe they are representing themselves in a prosocial manner on their online profile, forgetting or ignoring that this can conflict with their professional responsibilities. Nonetheless, any single incident of an online depiction represents the tension between personal pride in compassionate acts and unethical and potentially illegal representations and descriptions of individuals receiving medical care.

Medical mission trips offer an opportunity to trainees and doctors alike to learn to practice medicine outside of the highly technical US hospitals and to gain personal satisfaction in treating patients who may otherwise not have access to care. However, these acts of compassion or benevolence should not be available for public or private discussion or viewing outside the context of the doctor-patient relationship. Medical trainees and providers at all levels need to apply legal and ethical practices of patient privacy at all times of their working careers. We believe that photographs of patients from medical mission trips are unethical and unprofessional, yet, due to variances in established international and emerging Internet law, they are only a possible privacy violation.

HIPAA (1996) [7,8] and other laws such as the Health Information Technology for Economic and Clinical Health Act demonstrate that the legal aspects of protecting patient identities in the digital age are complex [16-18]. In this study, students and residents do not appear to violate patient privacy at their own US institutions through online postings, but they seem to not equate this standard to medical mission trips in other countries. The Hippocratic Oath, HIPAA, and individual state and international laws all articulate different regulatory standards of patient privacy to which health care providers, as “covered entities,” must adhere. While extensive and at times confusing, they are nonetheless the law. Medical mission trips within the United States, for example, would characterize written patient health information on the Internet as HIPAA violations [7,8], but potentially not a photograph if it is not a “full-face photographic image” [19]. Other countries, such as Argentina, have stricter patient privacy laws that may include any photography [20]. Further, state laws in the United States may dictate higher standards than the federal HIPAA law for their licensed practitioners. In Florida, for example, all physicians are required to always maintain patient confidentiality regardless of where they are. To date, there is no legal precedent for the adjudication of these potential online violations, nor guidance from the medical literature on how to maintain high standards of patient privacy in the age of online social networking. To the contrary, in fact, one publication (predating online user-generated content) *advocated* the use of digital photography, ostensibly for its ease of transmission and reproduction [21]. It is yet unknown who, outside of the individual patient, could claim a violation when viewing online content. Nonetheless, awaiting legal action is ill advised.

Like the legal aspects, the ethics involved are multifaceted. In speaking to the responsibilities of health care providers who place patient information online, social networking sites challenge the difference between public and private information. In fact, one might argue that, while these sites are public, users are likely operating under the expectations of privacy [22]. However, users of social networking sites not only choose to have profiles with photos, text, and other self-created content, they also have control over whether such content is available to everyone (publicly available) or whether their profile and its content are private to some or all. Of note, since this study was performed, Facebook has changed its privacy features (December 2009), requiring users to actively select what it describes as “simplified privacy settings.” However, its default settings allow for unrestricted public access, much to the consternation of Internet privacy and security experts [23]. It remains unknown how medical professionals will respond to this privacy option. Additionally, current academic discussions describe the exact nature of what is public versus private, or identified versus deidentified on the Internet as not dichotomous [24], and that privacy is ultimately a function of social context, meaning that displays and disclosure of information may be appropriate in some contexts but not in others [13]. Profiles and postings of any type—public or private—are ultimately the responsibility of the creators, who in this case are practicing medical trainees and/or professionals who have completed HIPAA and confidentiality training. Unique to the fields of health care, these roles and their attendant responsibilities continue beyond the end of a shift and into all spheres of their lives, including when traveling abroad.

Additional ethical considerations may question what duty that we, as authors, have in collecting and analyzing data obtained from public online social networking sites [12,13,16]. Foremost, as medical professionals, we are bound to report potential abuses of children [25]. We do not feel this has occurred. Additionally, it could be argued that research on social networking sites is voyeuristic, hence inappropriate. Leading researchers, however, have likened social network research to research on newspaper personal ads [26], removing much of the mystery surrounding its potential. We believe that medical educators need to be particularly sensitive to educating our students and residents about patient privacy with clear and salient guidance on the various aspects of professionalism as it pertains to online postings. Given the overwhelming popularity of social networking applications such as Facebook, and their convenient and compelling means by which to exchange personal information, educators must better inform students that posting patient information may lead to serious, unintended, and irreversible consequences.

### Practical Recommendations

We make the following recommendations. First, there should be a legal resource for physicians traveling on medical mission trips such as an online list of local laws, or a telephone legal contact. To our knowledge, this does not exist. Second, we believe institutions that organize medical mission trips should plan this type of ethics seminar prior to the departure of any trip, since the legal and ethical implications may not be intuitive. Further, while an understanding of local privacy laws prior to departure on a medical mission trip would be ideal, it is nonetheless, at minimum, advisable to be cautious and apply the strictest legal precedent to any situation. For example, physicians should never write any patient information in text form or use a full-face photograph of a patient receiving any treatments. If photographs of individuals are desired, written consent should be obtained (although the wording of such documents may still not be legally defensible in that country). Additionally, subjects should only be shown in profile or in shadows, or physicians/medical professionals should use photo editing software to deidentify patients’ faces (see Figures 2-5 for the authors’ examples of ways to deidentify patients and trainees). While photographs can play a central role for both physicians (eg, in dermatology) and patients (eg, the birth of a child), they are one of the most difficult legal and ethical considerations in online portrayals and as such demand careful attention.

### Study Limitations

This study has several limitations. First, this study was performed at a single institution, where it is possible that the students and residents with patient portrayals did in fact receive permission from the individuals that they photographed. However, no acknowledgement or supportive information regarding this consideration was available on the individual profiles. Second, while it appears that medical students are more likely than residents to post content that may violate patient privacy, this likely is a function of the structure of medical school in which students in their fourth year have the most time for trips overseas and their younger age [27]. Finally, we cannot comment on profiles that have been made private. It is likely, perhaps even more likely, that photographs or even text that may violate patient privacy exists on private sites, since profile owners may feel their audience is not public. However, given the large number of profile friends Facebook users have (sometimes thousands), the notion of privacy is again contextual [26]. Yet patient privacy is not contextual. It is concrete and unyielding to electronic and other innovations for social networking.

### Conclusions

As a profession, we have made considerable strides to protect patient privacy. We have not, however, adequately impressed upon students and residents that online social networking sites and blogs are, in essence, broad communities with a public audience. They are arenas, such as medical mission trips, in which patient information must be guarded just as it would be in any health care situation. Future studies should explore the motivations behind such postings, but we believe the observations found in this study merit swift action, since the nature of social networking sites allows for immediate assumptions by the observer, whether or not these assumptions are formed within the context that the profiler intended. Medical mission trips require the same high professional standards of patient privacy that all medical situations require, whether in a highly technical US tertiary care center or in a rural medical clinic in another country.

## Abbreviations

HIPAA: Health Information Portability and Accountability Act
